# Patient Interaction Involving Older Adults: Provider vs. Caregiver Expectations

**DOI:** 10.3390/geriatrics7050101

**Published:** 2022-09-17

**Authors:** Pooja Shah, Kaitlin Donovan, Robert Hubal

**Affiliations:** 1 Eshelman School of Pharmacy, University of North Carolina Chapel Hill, Chapel Hill, NC 27599, USA; 2 Renaissance Computing Institute, Chapel Hill, NC 27517, USA

**Keywords:** healthcare conversation, interaction best practices, older patients, caregiver expectations

## Abstract

This paper presents a study of the interaction between healthcare providers (HCPs) and older patients and their caregivers. The paper first presents results from a rapid review and narrative synthesis using PubMed and Google Scholar of HCP/patient/caregiver interactions involving older patients; these results then informed the design of a survey administered to HCPs and caregivers using a range of scenarios and their ratings of appropriateness of different responses, to explore where expectations align or differ between HCPs and caregivers. In analyzing ratings, the research found HCPs and caregivers generally approach the older adult encounter with similar expectations, but differences for specific situations are informative. HCPs appear to better recognize when there is a need to show empathy, as when a patient is frustrated or anxious. HCPs, overall, offer more calming responses, especially in embarrassing, upsetting, or worrying situations. For older patients of advanced age, HCPs value engagement with patients more than caregivers, but HCPs are more aligned with caregivers in their ratings of how to engage caregivers. Compared to caregivers, HCPs focus more on simplifying the description of treatment rather than using thorough explanations when a patient expresses hesitancy or avoidance. The results from this work suggest that having a fuller understanding of the different participants’ expectations may improve communication and identify potential pitfalls. A better understanding may also lead to changes in how students in the healthcare fields are trained; having better insight into this relationship will prepare them for interacting with older patients while addressing the needs of caregivers.

## 1. Introduction

As part of a series of studies conducted by the senior author investigating language use in clinical contexts [1,2,3], we conducted a study of healthcare provider (HCP) and caregiver expectations of older adult medical encounters. From their experience, HCPs have considerable understanding of processes that occur during interactions with older patients, but gaps remain in their delineating strategies and tactics to handle different situations. For instance, HCP beliefs about how an interaction should flow may or may not match those of patients or caregivers—with possible consequences regarding compliance with treatment. Similarly, the content or level of detail of the interaction may be perceived differently by HCPs and patients and caregivers depending on the situation. Further exploration of these gaps may inform HCPs on how to improve current patient encounters and improve the training of new HCPs. The focus of this paper is to study these gaps.

To gauge HCPs and caregivers’ expectations, we first conducted a rapid review and narrative synthesis [4] of the literature on recommended practices for HCPs during interactions with older patients and their families. Informed by that work, we then administered a survey to gauge HCP and caregiver expectations of interactions with older patients.

## 2. Rapid Review and Narrative Synthesis

The purpose of a synthesis of the literature was to inform the design of the surveys. Following a similar approach to our previous work [3], the review was intended to identify common elements of an encounter with an older patient and better understand the role of the caregiver. Though not as formal as a systematic review, we strove to consider a range of relevant studies that would allow us to develop realistic and varied scenarios for the survey.

### 2.1. Methods

We subjected PubMed and Google Scholar to a series of searches. For instance, we ran searches of {healthcare} {interaction} AND {age}, where the bracketed terms were instantiated with specific terminology (e.g., {healthcare} includes “healthcare” and “medical”; {age} includes “elderly patient”, “geriatric patient”, or “older patient”). From the results, we narrowed down the articles to those focused on HCP/older patient/caregiver interaction. We included English-only studies and, when possible, those reflecting more recent work. Our synthesis involved first reviewing abstracts to ensure that papers represented relevant content and then, for selected papers, carefully reading to extract main findings. We identified common themes across papers by noting recurring concepts and sought similarities among findings, especially where the situations presented in the studies differed.

### 2.2. Results

#### 2.2.1. Elements of the Encounter

Any successful healthcare-related encounter includes elements such as relationship building, information exchange, and empathizing with the patient [5]. Patients want HCPs to address their concerns, understand the “whole person” and social setting, and be understandable, approachable, and respectful [6]. Basic competencies (“entrustable professional activities” [7]) that HCPs need to demonstrate with their older patients include assisting them and their caregivers with medical decision making and providing age-appropriate consultation regarding treatment, medication management, and psychological and social concerns [8]. Older adult advocates have recently recognized the five “M’s” of age-friendly care, which include a focus on multi-complexity (chronic conditions, advanced illness, complicated biopsychosocial needs), mind, mobility, medications, and what matters most (meaningful health outcome goals and care preferences) [9].

#### 2.2.2. Influence of Caregivers

The encounters between HCPs and older patients and their caregivers reflect many similar elements to those between HCPs and pediatric patients and their parents [3]. Unlike pediatrics, though, older patients present with issues of futility, mortality, litigation, and possibly low prestige [10,11,12,13,14]. The dynamic is characterized as the “physician–elderly patient–companion triad” in an older adult medical encounter [15]. The third person in the dialog, generically the companion, but herein referred to as the caregiver [16], often controls the dialog during encounters and facilitates the conversation [17,18]. The caregiver can take on roles such as advocate for the patient, passive participant, or even antagonist [19,20]. There are “coalitions” that form naturally during these encounters [21], all in service of collaboration of care.

The role of the caregiver in an older adult medical encounter varies based on the five “M” patient characteristics [9]. For example, in settings with dementia patients and presumably also whenever patients are otherwise compromised, caregivers tend to expect HCPs to attend to them [22]. HCPs tend toward that focus of caregivers as they gain experience [23], though other tools are also useful in assessing cognitive deficits [24]. HCPs sharing treatment recommendations with caregivers can promote adherence [25], but this offer must be made in the context of partnership [26] and with clear expectations set [27]. A competency framework has emerged for caregivers that HCPs should support [28]. Improved triadic communication can result not only from better training of HCP knowledge and skills but also with a focus on the caregiver. For instance, caregivers’ health literacy influences their ability to navigate care for older patients [29]. For cancer in particular, and likely other disease states and chronic illnesses, as well as end-of-life discussions, there may be “concordance” between caregivers and HCPs and between patients and HCPs [30,31]; discord would suggest communication patterns calling for improvement. Preliminary evidence shows that different priorities set by HCPs versus patients and caregivers causes conflicts in treatment decisions [32] and different perceptions of how well HCPs communicate prognoses [33]. In all of these studies, there are gaps among expectations and outcomes for different participants in the encounter.

There are roles played by specific diseases (if any) in the relationship between an HCP and caregiver, the focus on informal versus formal caregiving, and legal representation (e.g., guardianship vs. friendship). Further, an HCP may or may not have the legal right to provide medical information to the caregiver, depending on the older patient’s agency. In the work presented below, we treat the relationship as that of a supporter—present to assist the older patient where or when needed and integrated into the healthcare process [34] but not necessarily to make medical decisions.

#### 2.2.3. Different Providers

“Healthcare provider” is a broad term that includes members of an interprofessional team (physicians, nurses, nursing assistants, pharmacists, specialists, social workers). The form and function of communication with caregivers and patients for each member necessarily differs. A meta-analysis showed that pharmacists’ role in team-based healthcare focuses on safety and adherence to treatment [35]. Core knowledge, skills, and attitudes (KSAs) may be needed in pharmacists’ training [36], although full implementation of these competencies may be lagging [37,38]. Dentists recognize age-related oral health considerations [39]. Mental health professionals address geriatric-specific concerns associated with chronic disease and cognitive decline [40,41]. Though family care physicians are on the front lines of medical assessment [42,43], a team approach is needed to maintain focus on accuracy [44], to identify cognitive impairment [45], and to avoid adverse drug events [46]. Overall, there is a shortage of HCPs; there are too few relative to the growing need [47,48].

#### 2.2.4. HCP Learning

Even a single home visit by novice HCPs, followed by reflection, has been found to lead to improved care [49]. Other researchers have elaborated on this reflection and found increased awareness of the need for family and community support and a focus on unique aspects of geriatrics, such as mortality and ethical/moral challenges [50,51]. Though somewhat dated, studies have found that a learning module helps novice HCPs (such as student providers) to understand issues associated with communication and ageism [52] and that these individuals can increase their understanding of physical decline and their comfort with eldercare issues [53]. Specific interventions, such as completing a knowledge test or taking the perspective of someone aging, differently affect novice HCPs’ attitudes and empathy toward older patients [54]. Some benefit may accrue by having these individuals engage in directed narratives, such as mind maps [55] or theater [56]. One literature review identified the potential of KSAs across multiple programs—coursework, mentoring, simulation, and clerkship [57].

## 3. Survey

These literature-based findings suggest a range of considerations for HCPs during encounters with older patients. What the studies do not directly address is how closely HCPs and caregivers align in different, often challenging situations, yet their accord can be important for patients’ compliance with treatment. The goal of this part of the study was to identify situations with common understanding between HCPs and caregivers and those with mismatching ideas of how best to proceed with the geriatric encounter, with the intent of deriving best practices that can inform HCPs’ actions.

### 3.1. Methods

#### 3.1.1. Materials Development

We developed a survey to gauge HCP and caregiver expectations of interactions with older patients. We started by creating scenarios, generated from case studies, the rapid review and narrative synthesis, and the first author’s clinical experience. Based on vignette analyses from medical education [2], we structured scenarios using the following template:

[Initials] is a [age]-year-old [gender] who presents to [setting] with a chief complaint of [medical condition]. [Sentences about condition and symptoms, controlled or not]. [Statement about HCP imperative in the current situation].

Scenarios involved topics such as required changing of a patient’s long-used medication, pain and dependence on pain medication, new or worsening diagnosis, a necessary complex procedure or surgery, proper adherence to medication therapy, cost of medications and insurance, and disability. We evenly spread ages and genders of patients across scenarios.

A total of 28 scenarios—14 having female patients and a matching 14 having male patients—were targeted toward HCPs (see Table S1 in Supplementary Materials). We adapted these scenarios into a parallel set for caregivers. First, we kept all details, but replaced descriptions of the patient (“a 66-year-old female”, “presenting to your practice…with his daughter, who is his primary caregiver”) with personal references (“You are a 66 year-old female”; “visiting the practice with your daughter, who takes care of you”). Second, details or terminology that caregivers might not know (“complaint of neuropathic pain”) were replaced with more common language (“shooting pain and tingling in the hands and feet”).

As we developed scenarios, we determined response options. Each pair of scenarios had a goal, determined by the topic. For instance, for the topic of adherence to medication, responses related to educating the patient on the importance of adherence. Similarly, for the topic of presenting a needed complex procedure, the responses related to discussing that need with patients. All responses were given using a sliding scale (slider bars provide visual cues and have been shown to be generally as effective as traditional Likert scales [58]) from zero to 100, in response to the prompt, “Slide the marker to indicate how strongly you feel (0 = should not happen; 100 = should definitely happen) that ___ should take the action during the examination”, where “___” was “you” for HCP respondents and “the HCP” for caregivers. As was logical, we presented the same response set for each scenario.

We created two non-overlapping surveys, carefully arranging equivalent mixes of scenario types and patient ages and genders between survey variants. To avoid order effects, a given participant answered questions for each of the 14 scenarios, presented in random order; the choice of survey variant was administered was also randomized, as were response options.

#### 3.1.2. Scenario Characteristics

Our intent was to seek expectations from HCPs and from caregivers of older patients about appropriate strategies when HCPs interact with older patients across different situations. We called these situations “scenarios” and characterized them using three overlapping criteria that reflected the content presented by, the tasks inherent in, and those individuals involved in the scenarios [59]:

1. Content of scenario itself. We categorized scenarios into seven non-mutually exclusive buckets: those (i) requiring focused *discussion* or *explanation*; (ii)–(v) for which patients might be expected to be *anxious*, *embarrassed*, *sad*, *upset* or *frustrated*; (vi) for which patients might be trying to *avoid medical care*; and (vii) involving *urgency*.
2. Type of responses being asked of HCP or caregivers. Following our prior work, we derived 15 non-mutually-exclusive buckets (see Table S2 in Supplementary Materials): those responses that (i) queried about an ability or need for *communication* with the older patient in the situation; (ii) involved perceptions of need for *empathy*; (iii) addressed *strategies* for dealing with the older patient; (iv) addressed *tactics* (more low-level actions than the general approach addressed by strategies); (v) focused on HCPs portraying *calm demeanor*; (vi) focused on HCPs providing *comfort*; (vii) focused on HCPs being *matter-of-fact*; (viii) focused on HCPs providing *explanation*; (ix) asked about any medical *education*; (x) focused on HCPs demonstrating and maintaining *control*; (xi) gauged *caregiver engagement*; (xii) gauged *patient engagement*; (xiii) gauged *reliance on others* in the healthcare setting; (xiv) indicated a need for HCPs to *simplify* the process; or (xv) indicated a need for HCPs to ensure *thoroughness* of the process. We validated the assignments through a convenience group of 28 participants via Mechanical Turk (mTurk); an assignment of a response to a bucket was made only if agreed to by most (defined as greater than the median) participants.
3. Ages of patients in scenarios. Though any cutoff is somewhat arbitrary, patients generally rely more on caregivers as they age. We chose over-65, a common if not universal starting point for older patients, and used ages (i) 66–70, (ii) 71–75, (iii) 76–80, (iv) 81–85, and (v) 86–90.

#### 3.1.3. Procedures

Participants. There were 51 HCPs and 50 caregivers. Participants were recruited using mTurk, an online service allowing enrollment in studies of individuals having self-identified, verified backgrounds. We required individuals to be employed in the healthcare industry when responding to the HCP survey or caring for patients 65 and older when responding to the caregiver survey. There was no age limit or other restriction on caregivers.

Survey Administration. Surveys were administered using Qualtrics (Provo, UT, USA). Each participant who was vetted as an HCP or a caregiver was sent a unique link through the mTurk service, presenting one randomly selected block of scenarios. Within the block, scenarios were presented randomly. Qualtrics automatically collected and labeled participant responses.

Statistical Analyses. Data were downloaded from Qualtrics and analyzed using the R statistical package (www.r-project.org; accessed 22 May 2022). Aside from descriptive statistics (numbers, means), analyses were conducted using two-way analysis of variance to identify the influence of variables of interest for the particular research question. We report F statistics of differences in population means and consider an individual test to be significant at *p* < 0.05.

Ethical Review. The study was reviewed and exempted by an Institutional Review Board at our institution.

### 3.2. Results

On average, participants required just under ten minutes to complete the survey, suggesting a low burden. Table 1 and Table 2 provide summary statistics on respondent characteristics. Though not all participants will have had experience with all scenarios presented, in these analyses, data from all healthcare respondents were pooled, and the same was done for caregivers.

Analysis showed no effect of survey variant on respondent ratings (F(1, 89) < 1, *ns*) nor any interaction with source of participants (HCPs, caregivers; F(1, 87) < 1, *ns*), so all responses were pooled.

#### Planned Research Questions

The ratings scale asked respondents to indicate how strongly they felt that each given response should (100) or should not (0) occur. Overall, the source affected the ratings—HCPs (72.4) versus caregivers (57.8) (F(1, 89) = 16.8, *p* < 0.0001)—mirroring our previous findings of higher HCP ratings across scenarios [3]. However, this average difference changed when planned queries were applied.

To identify how HCPs and caregivers differ in their approach to or expectations of a situation (based on values they assigned to different responses to different scenarios), we queried the data to address a number of research questions.

**Figure 1:**
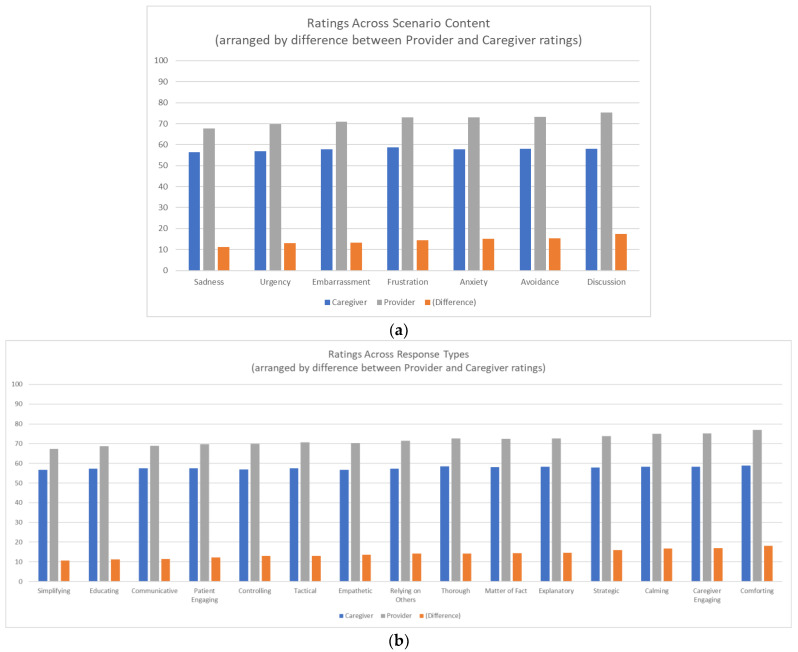
(**a**) Ratings for scenarios across different scenario content; (**b**) Ratings for scenarios across different response types. Note: Content and response types are arranged by the difference between HCP and caregiver ratings; all differences themselves are significant (F statistic *p* < 0.05).

To begin, we asked if HCPs and caregivers differ in how to handle different situations. We found no main effect of content of the scenario; however, there were significant interactions between HCPs and caregivers for all different scenario contents (Figure 1a) and for all different response types (Figure 1b). Planned contrasts described next investigated how HCP and caregiver ratings are closer for certain scenario and response combinations than they are for others.

First we considered if HCPs and caregivers differ for discourse (involving communicative or empathetic response) versus active responses (strategic or tactical actions). The data show a large effect between HCPs and caregivers, where discourse ratings rise more over active ratings for HCPs than for caregivers. A similar pattern holds for which ratings rise more for comforting responses than for controlling responses, for scenarios involving discussion compared to urgency, and for both explanatory and thorough responses over simplifying responses for scenarios requiring focused discourse. Similarly, we considered if HCPs and caregivers differ for scenarios that require communication, for responses that are explanatory, thorough, or simplifying. The data show that HCPs value both explanation and thoroughness over simplification more than caregivers do. Additionally, for scenarios that involve patients avoiding versus not avoiding treatment, the data suggest HCPs value more thorough responses for avoidance scenarios than non-avoidance scenarios than do caregivers, though not for simplified, controlling, explanatory, educating, or matter-of-fact responses.

Next we considered scenarios involving affect. HCPs, relative to caregivers, value thorough responses for worry/anxiety scenarios more so than simplifying responses, compared to those scenarios that do not involve anxiety. HCPs value comforting and more engaging responses, as well as calming responses, more so in scenarios that do not involve sadness than those that do. HCPs value comforting responses for upset/frustration scenarios more so than caregivers compared to scenarios that do not involve upset/frustration. HCPs value simplifying and even more so thorough responses, compared to caregivers, in scenarios that do not involve sadness than scenarios that do. However, HCPs do not value comforting responses for embarrassment scenarios more than caregivers compared to those scenarios that do not involve embarrassment, nor matter-of-fact or calming responses.

**Figure 2:**
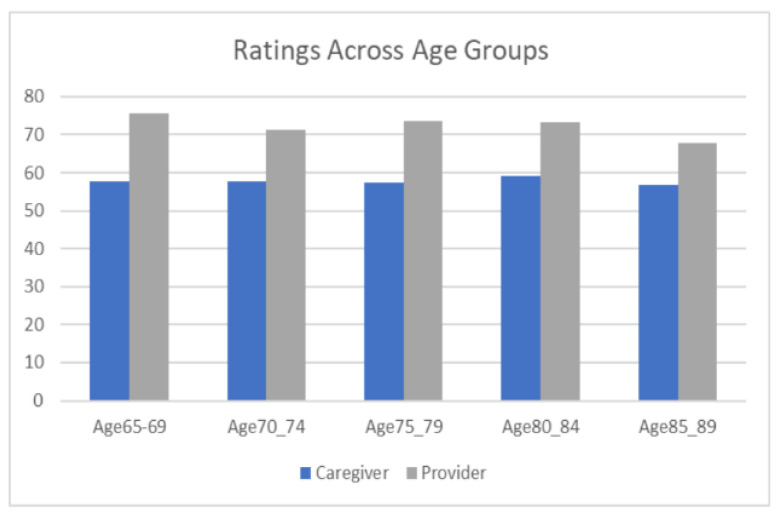
Ratings for scenarios as patients’ age range increases.

Finally we considered managing patients of different ages. There was no main effect of age of patient in the scenario, but a significant trend toward a narrower gap between HCPs and caregivers (Figure 2). Specifically for engagement with the youngest (66–70 year old patients) and oldest (86–90) age groups, the data show caregiver ratings are equivalent for both age groups, but HCP ratings are higher for the youngest age group than the oldest. This finding is true for specific response types such as education, thoroughness, and patient engagement, though engagement with the caregiver increases more for the older age group than the younger age group. Relatedly, we considered if HCPs and caregivers differ in how they view participants in the scenario. Caregivers rate caregiver interactions equally with patient interactions, whereas HCPs rate caregiver interactions as more important than patient interactions.

## 4. Discussion

The results suggest that HCPs understand or are willing to provide additional support to patients, in comparison to caregivers, in situations (such as anxiety scenarios) where simplifying responses are important or for situations (such as embarrassment) where matter-of-factness is needed. Different situations call for greater focus on specific responses, whether comfort, calming, or explanation. We reasoned HCPs should be more aware of scenario-specific needs than caregivers. The data bear this reasoning out; there is a difference between HCPs and caregivers for different scenarios where specific types of responses are warranted.

Regarding a difference between HCPs and caregivers, across all scenarios, for specific types of responses, we reasoned HCPs would tend to value more detailed and matter-of-fact information exchange, given their greater medical knowledge, and caregivers less detailed and more explanatory or comforting information. The data show, however, that HCPs valued comforting responses over control responses, suggesting that they are aware of the needs of caregivers. Further, we expected HCPs and caregivers to value calming, comforting, and empathetic responses equally. The data show this expectation mostly holds, though HCPs rate calming, comforting, and empathetic responses less relevant for urgent scenarios, whereas caregivers show no differences among scenarios. In contrast, for explanatory, matter-of-fact, and thorough responses, there are few differences for HCPs across scenarios, in line with caregiver ratings.

Finally, regarding managing patients of different ages, we identified a trend toward a narrower gap between HCPs and caregivers, suggesting they are more in line with each other as patients age. HCP ratings generally declined as patients in the scenarios aged, although this finding may be a consequence of the content of those scenarios, with older patients experiencing worse medical situations. Nevertheless, the pattern did not hold for all types of responses; matter-of-fact and empathetic responses, for instance, were rated by HCPs about the same across patient age groups. We also looked specifically at engagement with the youngest and oldest age groups; we reasoned HCPs and caregivers would be more in line with a younger age group and differ in perceived needs from an older age group. The data support this reasoning. For scenarios that involve patients aged 86–90, we expected HCPs to see the same need to engage with caregivers as caregivers do, but a greater need to engage with patients. The data suggest HCPs significantly value engagement with patients for this age group more than caregivers. However, HCPs and caregivers value engagement with caregivers equally.

### Limitations

There is a range of roles for caregivers that are not fully addressed in this work, including supportive tasks like medication monitoring, transportation, and providing companionship. There are also differing views of division of responsibilities for those roles between HCPs and caregivers [60]. Further, our sampling method using mTurk likely did not lead to a representative sample of all types of HCPs or caregivers. In particular, in line with other studies [61], physicians under-participated in this survey (Table 1), and as well we note that fewer caregivers of the most elderly patients responded (Table 2). Additional series of scenarios with a wider array of participants may identify other differences in expectations between HCPs and caregivers. Finally, to allow for parallel methodology with our pediatric study [3], patients were not surveyed in this study, so their opinions on their own care were not directly taken into account.

## 5. Conclusions

HCPs and caregivers are not so different in how they perceive the geriatric encounter, with some exceptions. HCPs’ overall ratings were higher than caregivers, but this finding might be due to HCPs being more focused on the interaction with older patients than caregivers, or to a different interpretation of the instructions given. However, differences for specific situations are informative.

HCPs value being calm throughout an interaction, even more so than caregivers. HCPs value providing simple explanations when warranted, more so than caregivers, as in situations where patients are hesitant toward or avoid treatment or where they are embarrassed or anxious. HCPs are more attuned than caregivers to situations involving “younger” older patients, but HCPs and caregivers align as patients in the situations age. Even in those situations with much older patients, HCPs place greater value on addressing patients than do caregivers, though HCPs and caregivers place equal emphasis on addressing caregivers.

### Practical Implications

HCPs, patients, and caregivers all value participation in research endeavors, though they bring different perspectives of application [62]. There is thus reason to believe education and training following this work will have value. Training involves presenting situations for students to engage in; the more realistic the assessment of performance in those situations, the better transfer that is expected [63]. A goal is to develop a suite of geriatric cases to allow students to hone their KSAs to meet expectations of their older patients and their caregivers. We also expect there are other unique populations to be considered, including people with special needs and underserved communities, to identify gaps in how interactions are perceived by HCPs and those patients and their caregivers.

## Figures and Tables

**Table 1:** Participant characteristics—healthcare providers.

**Occupation**	**N**
Nurse, PA	20
Pharmacist	14
Physician	7
Mental Health	5
Technician	2
n/a	3
**Practice Setting**	**N**
Community Pharmacy	1
Hospital (ER)	5
Hospital (Inpatient)	16
Primary Care (Outpatient)	18
Specialty (Outpatient)	6
Urgent Care	3
Unspecified	2
**Percentage of Work Involving Older Patients**	65.4%
**Years of Practice**	**N**
<1 Year	1
1–3 Years	17
4–10 Years	10
>10 Years	21
Unspecified	2

**Table 2:** Participant characteristics—caregivers.

**Age of Person Cared for by Respondent**	N *
60+ Years Old	18
70+ Years Old	18
80+ Years Old	12
90+ Years Old	4
**Age of Respondent**	**N**
<30 Years Old	12
31–59 Years Old	31
60+ Years Old	7

* Some respondents were caregivers to multiple patients.

## Supplementary Materials

The following supporting information can be downloaded at: https://www.mdpi.com/article/10.3390/geriatrics7050101/s1, Table S1: Scenarios. (Listed by content, but presented to participants in random order). Table S2: Response options and their mappings. (Alphabetical, but presented to participants in random order).
