# Supplementary material for: Patient Interaction Involving Older Adults: Provider vs. Caregiver Expectations
Source: Geriatrics (Basel). 2022 Sep 17;7(5):101. doi: 10.3390/geriatrics7050101 (PMC9498497; doi:10.3390/geriatrics7050101)
Supplement: Supplementary file 1 [file geriatrics-07-00101-s001.zip › geriatrics-1778557-supplementary.pdf]

geriatrics

Article

Patient Interaction Involving Older Adults: Provider vs. Caregiver Expectations

Pooja Shah [1], Kaitlin Donovan [1] and Robert Hubal [2,*]

[1] The University of North Carolina Chapel Hill Eshelman School of Pharmacy, Chapel Hill, NC, 27599 USA
[2] Renaissance Computing Institute, Chapel Hill, NC, 27517 USA
* Correspondence: hubal@unc.edu; Tel.: +1-762-233-1763

Table S1. Scenarios. (Listed by content, but presented to participants in random order)

Scenario #	Scenario Content	Scenario (Participant Group A)	Scenario (Participant Group B)	Instructions (refer to Table A2 for list of responses)	Characteristics
9	Adherence to Medication	HW is an 80 year-old female who has been diagnosed with type 2 diabetes several years prior. She has come to the practice for a routine physical where she reveals that she only takes her prescribed metformin on days that she finds her blood sugar to be over 250 mg/dL. She claims that depending on the day, she sometimes takes a single pill, two pills, or doesn't take medication at all. Despite her lab results, she is insistent with you that her form of therapy works. Based on her history, it is likely that she will need to be prescribed metformin 500mg tablets to take twice per day. You will need to reassure the patient of this approach and discuss compliance.	GW is a 77 year-old male who has been diagnosed with hypertension several years prior, which is when he was prescribed to take Lisinopril. He has come to the practice for a routine physical where he confides that he does not take his medication regularly. He reveals that because he only takes Lisinopril when his blood pressure readings exceed 140/90, he often skips doses, or only takes half doses. Despite lab findings, he insists that this approach works fine for him. You believe that GW should be prescribed Lisinopril 20mg tablets to be taken once daily. You will need to reassure the patient of this change in approach and discuss compliance.	How would you go about educating to the patient the importance of adherence to their medication? For each, slide the marker to indicate how strongly you feel (0=should not happen; 100=should definitely happen) that you should take the action during the encounter.	Avoidance, Discussion, Urgency
12	Assistive Devices	BW is an 82 year-old female who has come to your practice with a chief complaint of dry eyes. She divulges that she has recently developed arthritis in her hands, and her joint pain is making it difficult to use her eye drops. Her dry eyes have become very uncomfortable and for the last five years she has been using eye drops to manage it. She is embarrassed with this situation as she can no longer treat herself. You believe she may benefit from the use of an assistance device such as AutoSqueeze, though it will be	SJ is an 81 year-old male who has come to your practice with a chief complaint of dry eyes. He divulges that he has recently developed Parkinson's disease and his tremors are making it difficult to use his eye drops. His dry eyes have become very uncomfortable and for the last five years he has been using eye drops to manage it. He is embarrassed with this situation as he can no longer treat himself. You believe he may benefit from the use of an assistance device such as AutoDrop, though it	How would you explain the need for the patient to use an assistive device to help continue her self-treatment? For each, slide the marker to indicate how strongly you feel (0=should not happen; 100=should definitely happen) that you should take the action during the encounter.	Discussion, Embarrassment

Table S1. Scenarios. (Listed by content, but presented to participants in random order)

Scenario #	Scenario Content	Scenario (Participant Group A)	Scenario (Participant Group B)	Instructions (refer to Table A2 for list of responses)	Characteristics
		noticeable to others when she uses it.	will be noticeable to others when he uses it.		
1	Changing Long Term Medication	BC is a 80 year old male who presents to your practice for an annual checkup. His past medical history includes anxiety, hypertension, and type 2 diabetes. During the encounter, you find out that he has had two falls in the last year and feels more unstable recently. Upon reviewing his medications you find that he is currently taking lorazepam for his anxiety, and he feels that the lorazepam works well for him. He once tried taking some herbal supplements that he was told would relieve anxiety, but says that they did not improve his condition, so he is hesitant to change, because any change in his treatment regimen contributes to his anxiety. He also feels that the falls were unrelated to any medication. But due to his advanced age and recent increase in falls, you believe it may be safer for him to switch to sertraline.	CJ is a 76 year-old female who presents to your practice for an annual checkup. Her past medical history includes insomnia, hypertension, and type 2 diabetes. During the encounter, you find out that she has had two falls in the last year and feels more unstable recently. Upon reviewing her medications you find that she is currently taking alprazolam for her insomnia, and she tells you that her alprazolam works great for her. When asked about her past medications for insomnia she recalls trying melatonin many years ago, but she says that it did not improve her condition, so she is hesitant to change to any medication that makes her more tired than she already is. She also feels that the falls were unrelated to any medication. But due to her advanced age and recent increase in falls, you believe it may be safer for her to switch to trazodone.	How would you go about discussing the new medication and the need for a change in medication with the patient? For each, slide the marker to indicate how strongly you feel (0=should not happen; 100=should definitely happen) that you should take the action during the encounter.	Anxiety, Avoidance
6	Complex Procedure	BR is a 70 year-old female who presents to your practice with the chief complaint of chronic knee pain. Her knee pain has lasted for many years and affected her daily activities as well as exercise. She has a previous medical history of osteoarthritis. BR has been attending physical therapy for over a year and has tried a variety of pain medications. Her knee pain still seems to be worsening and has previously sustained substantial damage caused by a severe injury. She is upset because she has always been very active and the pain and injuries have severely curtailed her activity. It is necessary to perform a procedure to remove damaged parts of the knee and replace it with metal and plastic pieces to restore function. You must	MW is a 68 year-old male with a past medical history of osteoarthritis. He presents to the practice after having chronic hip pain that has begun interrupting his daily activities and precluding exercise, as he can no longer deal with the pain. He has tried physical therapy and a variety of pain medications to relieve his symptoms, but nothing has worked. MW is upset because he is no longer able to run or even swim in comfort. His left hip has sustained substantial damage, and it is clear that he needs surgery to remove the damage parts and replace them with metal or plastic pieces to restore function. You must discuss with the patient the extensive recovery and	How would you go about discussing the difficult procedure with the patient? For each, slide the marker to indicate how strongly you feel (0=should not happen; 100=should definitely happen) that you should take the action during the encounter.	Discussion, Frustration

Table S1. Scenarios. (Listed by content, but presented to participants in random order)

Scenario #	Scenario Content	Scenario (Participant Group A)	Scenario (Participant Group B)	Instructions (refer to Table A2 for list of responses)	Characteristics
		discuss with the patient how long the recovery will be and what form of rehabilitation it will take.	rehabilitation required after the surgery.		
11	Demonstration on How to Use Medication	VJ is a 66 year-old female who has come to your practice with the chief complaint that her blood sugar has become more difficult to control with her metformin. She was diagnosed with type 2 diabetes several years ago and has been treating it diligently with her prescribed metformin, however, this treatment is no longer controlling her condition. You believe that prescribing her Ozempic to be taken weekly may allow for VJ to have better control over her diabetes. Ozempic must be administered subcutaneously with a pen, however, and VJ is anxious about injecting herself as she has never self-injected previously. You must assure the patient that she will be able to comply with this regimen.	MN is a 69 year-old male who currently suffers from rheumatoid arthritis. This has been going on for five years. He has come to your practice with complaints that he often still has joint pain and stiffness. MN has been taking methotrexate since his initial diagnosis however due to disease progression, monotherapy methotrexate is no longer enough. You believe that it is in MN's best interest to additionally prescribe Humira to be biweekly and subcutaneously injected with a pen to further manage his symptoms. However, MN is anxious of injecting himself as he has never self-injected previously. You must assure the patient that he will be able to comply with this treatment.	How would you go about the situation if the patient were reluctant to self administer the pen? For each, slide the marker to indicate how strongly you feel (0=should not happen; 100=should definitely happen) that you should take the action during the encounter.	Anxiety, Discussion
13	Deteriorating Senses	JP is a 78 year-old male who has presented to your practice for a follow up for hearing loss. JP doesn't believe it is really a problem, but his partner has noticed that he has difficulty conducting conversations over the phone and prefers the volume on the television to be exceedingly loud. JP's partner would like for him to receive a thorough check up and auditory testing. JP's partner is worried that his lack of urgency within the matter is concerning and it is affecting the quality of JP's life. You believe it is important to evaluate JP for hearing loss and potentially make an appointment for a hearing aid fitting, and need to reassure the patient that this is the best plan.	WM is a 79 year-old female who has presented to the office for a follow up appointment regarding her vision loss. She currently wears glasses and has suffered from poor near-sighted vision for the last 20 years. Most recently, WM has been having trouble reading things. She doesn't believe it is really a problem, but her partner would like for WM to get a thorough check up and vision test. WM's partner believes her denial and poor vision is seriously impacting her quality of life. You believe it is necessary to evaluate WM for far sighted vision loss and potentially upgrade her glasses to bifocals, and need to reassure the patient that this is the best plan.	If the patient is not agreeing to {auditory/vision} testing, how do you go about discussing with the patient the need for further testing and if any item such as cost was playing a role? For each, slide the marker to indicate how strongly you feel (0=should not happen; 100=should definitely happen) that you should take the action during the encounter.	Avoidance, Discussion
3	New Diagnosis	KC is an 85 year-old female who presents to your practice with a chief complaint of urinary urgency and frequency.	DF is an 82 year-old male who presents to the practice with chief complaints heart palpitations and shortness of breath.	How would you go about discussing the diagnosis with the patient? For each, slide the marker to indicate	Anxiety, Embarrassment

Table S1. Scenarios. (Listed by content, but presented to participants in random order)

Scenario #	Scenario Content	Scenario (Participant Group A)	Scenario (Participant Group B)	Instructions (refer to Table A2 for list of responses)	Characteristics
		She has complaints of having a strong sudden desire to urinate and recently has been waking up almost every night to urinate. The urgency started about a year ago, but recently has gotten much worse with the most recent symptoms being needing to go often in the middle of the day. She is not sure what is going on and is very sensitive about talking about the topic because she is worried that she may start wetting herself or her bed. Due to the gradual progression and lack of other symptoms like pain or hematuria you make a diagnosis of overactive bladder.	He has complaints of feeling his chest thump especially as he is walking about during the day that cause him to temporarily stop what he is doing. Though he has had symptoms for a few years, they have become far more prominent in the last couple of weeks. He is worried about what is going on and is sensitive to talking about it since his worsened symptoms have begun to affect his daily life and stress level. After examination, it is clear that he has an irregular pulse, and you suspect an ECG will be consistent with a diagnosis of atrial fibrillation.	how strongly you feel (0=should not happen; 100=should definitely happen) that you should take the action during the encounter.	
4	New Diagnosis	RD is a 74 year-old male who presents to your practice with chief complaints of joint pain and swelling in his hands. He tells you that not only does he sometimes have extreme pain in his fingers on his left hand, but also he suffers from stiffness. He is worried because of his lack of mobility, and fears that he will be unable to live independently. A physical examination is performed and indicates a decreased range of motion and tenderness in the affected joints on his left hand. Due to RD's asymmetrical joint involvement and lack of fluid buildup around his joints, you believe he is suffering from osteoarthritis, and wish to discuss treatment and therapy options.	TS is a 71 year-old female who presents to your practice for a follow up appointment after recent hospitalization for a fragility fracture. The fracture, located in her hip, was the result of a fall. Her lack of stability worries her, and she fears that she will be unable to live independently. A DXA scan is performed and indicates that she has a T-score of -3, which is consistent with a diagnosis of osteoporosis. Given TS's advanced age, sex, and medical history including past fractures, you wish to discuss with her medical options for osteoporosis.	If the patient were not covered by health insurance (and never even paid Medicare taxes), how would you discuss treatment options? For each, slide the marker to indicate how strongly you feel (0=should not happen; 100=should definitely happen) that you should take the action during the encounter.	Anxiety, Discussion
7	New Life-Altering Diagnosis	DH is an 89 year-old male who has been brought to the practice by his caregiver with the chief complaint of uncontrollable muscle movement. He has had tremors for a year, but they have recently worsened and begun disturbing his daily life. He often has difficulty holding objects. The caregiver also reports that DH has a forward tilt while walking. Based on his symptoms, it seems that DH is suffering from Parkinson's. After informing DH and caregiver of	WS is a 87 year-old female who has been brought to the practice by her caregiver with chief complaints of memory loss that has been affecting her daily life. WS complains of having a forgetful memory for the past few years, but within the last six months the memory loss has become more severe. Her caregiver notes that she has additionally developed behavioral problems including a lack of patience, a quick temper, and	How would you go about discussing this life altering diagnosis given that the patient is visibly upset? For each, slide the marker to indicate how strongly you feel (0=should not happen; 100=should definitely happen) that you should take the action during the encounter.	Frustration, Sadness

Table S1. Scenarios. (Listed by content, but presented to participants in random order)

Scenario #	Scenario Content	Scenario (Participant Group A)	Scenario (Participant Group B)	Instructions (refer to Table A2 for list of responses)	Characteristics
		the diagnosis, you notice the patient has become visibly upset and emotional.	days with a low mood. After referring to the DSM-5 criteria and MMSE screening tool, it seems that WS has mild dementia. After informing WS and caregiver of the diagnosis, you notice the patient has become visibly upset and emotional.		
5	Outpatient Procedure	RF is an 83 year-old male who presents to the practice after previously developing a reddish brown lump on his neck two weeks ago. It first appeared as a small freckle, but within the past few days has grown in size and become asymmetrical. RF is distressed by the look of the lump. Upon further questioning, he admits that he took a long beach trip with his wife about a month ago, and he didn't purchase or wear any sunscreen after running out. RF denies having any pain or changes in health status, and is upset both with the lump and that his wife is making him deal with the lump. Labs come back negative, and he has no palpable lymph nodes. You believe this to be a local cutaneous melanoma and an excisional biopsy is needed to remove the melanoma and complete further testing.	GJ is an 84 year-old female who presents to the practice with a chief complaint of blurry and deteriorating vision. She admits that she becomes frequently agitated because the cloudiness of her vision has given her great difficulty seeing at night. She is having increasing trouble completing her day to day activities, and is upset that she is forced to deal with her eyesight. Upon examination of her eyes, it is noted that there are cataracts located in both eyes. The lenses on each of her eyes have a whitish cloud which is impacting her vision. You believe GJ's best option for achieving better eyesight is to have cataract surgery and then follow her to gauge effectiveness.	How would you go about explaining to the patient the need for an excisional biopsy? For each, slide the marker to indicate how strongly you feel (0=should not happen; 100=should definitely happen) that you should take the action during the encounter.	Avoidance Frustration
10	Switching Medications from Brand to Generic Due to Insurance	LT is a 74 year-old female who has come to your practice with the chief complaint about her asthma inhaler. She has been using the Proventil HFA inhaler for the last seven years, but last week when she picked up her inhaler refill her insurance no longer covered Proventil and instead she picked up the generic albuterol sulfate HFA. LT is very upset about this switch as she has concerns about its efficacy. You feel that the switch from the brand to generic is still safe and efficacious for the patient's asthma, and must assure her that is the case.	TS is a 73 year-old male who has come to your practice with the chief complaint about his insulin pen. He has been using the Humalog Kwikpen for the last few years, but last week when he picked up his insulin refill his insurance no longer covered Humalog and instead he picked up the generic insulin lispro Kwikpen. TS is very upset about this switch as he has concerns about its efficacy. You feel that the switch from the brand to generic is still safe and efficacious for the patient's diabetes, and must assure him that is the case.	How would you go about educating the patient about the switch from a brand to generic medication? For each, slide the marker to indicate how strongly you feel (0=should not happen; 100=should definitely happen) that you should take the action during the encounter.	Avoidance, Discussion, Frustration

Table S1. Scenarios. (Listed by content, but presented to participants in random order)

Scenario #	Scenario Content	Scenario (Participant Group A)	Scenario (Participant Group B)	Instructions (refer to Table A2 for list of responses)	Characteristics
2	Switching Off of Opioids	WB is a 72 year-old male who presents to your practice with a chief complaint of neuropathic pain. He has had neuropathic pain in his hands and feet secondary to his type 2 diabetes for the past nine months. He first tried using over the counter naproxen with very little help. He then went to urgent care and was prescribed hydrocodone/acetaminophen for the pain. He feels that it works better than the naproxen, and even though he still has some nerve pain and tingling, he likes the overall feeling he gets from the opioid. WB would like to have an increase in his hydrocodone/acetaminophen dose for better control, and he is hesitant to try new medications. As his pain is neuropathic in nature, and to avoid any possible addiction or tolerance, you believe it may be in WB's best interest to switch off of opioids and change to gabapentin instead as this has shown better benefit for neuropathic pain and is safer than using opioids.	KD is a 75 year old female who presents to your practice with a chief complaint of neuropathic pain. She has had neuropathic pain in her feet and legs secondary to her type 2 diabetes for the past eight months. She first tried using over the counter ibuprofen with very little help. She then went to urgent care and was prescribed oxycodone for the pain. She feels that it works better than the ibuprofen, and though she still has some nerve pain and tingling in her legs, she likes the feeling she gets from the opioid. KD would like to have an increase in her oxycodone dose for better control, and she is hesitant to try new medications. As her pain is neuropathic in nature, and to avoid any possible addiction or tolerance, you believe it may be in KD's best interest to switch off of opioids, and change to duloxetine instead as this has shown better benefit for neuropathic pain and is safer than using opioids.	How would you go about discussing the new medication and the need for transitioning off of opioids with the patient? For each, slide the marker to indicate how strongly you feel (0=should not happen; 100=should definitely happen) that you should take the action during the encounter.	Discussion, Urgency
14	Worsening Common Condition	RS is a 66 year-old female who has presented to your practice with the chief complaint that her diabetes is worsening. She has had type 2 diabetes for the last 11 years and she is currently taking 13 medications for her multiple comorbidities. RS has mostly controlled her diabetes through taking metformin and glimepiride. Over the last year her blood glucose readings and A1c have been above goal despite her diligent medication use. You believe it is in the patient's best interest to add on empagliflozin to more effectively control her diabetes. However, RS is discouraged and hesitant to add on any new medications because she is already taking several, and because of the high cost of empaglifozin.	HM is a 67 year-old male who has presented to your practice with complaints of worsening hypertension. He has had hypertension for the last 12 years, and has been controlling his blood pressure with lisinopril and amlodipine. HM also has several other comorbidities for which he takes a total of 14 medications. Over the last year, his blood pressure readings have remained elevated despite medication use. You believe it is in the patient's best interest to add on a third medication, spironolactone. The patient is discouraged and hesitant to try a new medication because he is already taking several.	How would you explain to the patient that their condition may pose a legitimate threat to their life if it is not corrected with a new medication? For each, slide the marker to indicate how strongly you feel (0=should not happen; 100=should definitely happen) that you should take the action during the encounter.	Anxiety, Avoidance

Table S1. Scenarios. (Listed by content, but presented to participants in random order)

Scenario #	Scenario Content	Scenario (Participant Group A)	Scenario (Participant Group B)	Instructions (refer to Table A2 for list of responses)	Characteristics
8	Worsening Condition	LA is a 88 year old male who is presenting to your practice for a follow up on his lung cancer with his daughter, who is his primary caregiver. He used to smoke very heavily in his youth, but has tried to cut back over the years. He does not regularly keep up with yearly check ups and was hospitalized for a severe cough with bloody sputum over a year ago. There he was diagnosed with small cell lung cancer, and was treated with chemo and radiation. LA went into remission for a year; however, his cancer has returned and since metastasized to the liver. Due to the complexity of his condition and his advanced age, you believe that this patient's condition is likely terminal.	AS is a 86 year old female who is presenting to your practice for a follow up on her breast cancer with her son, who is her primary caregiver. The patient was initially diagnosed with breast cancer 2 years ago. During this time she was treated with a lumpectomy followed by radiation. Soon after she reached remission and now follows up her oncologist twice a year. During this follow up visit, her provider has found a metastatic recurrence of her cancer in her lungs. After further testing of her metastatic cancer, it was found to be triple negative breast cancer. Due to the complexity of her condition and her advanced age, you believe that this patient's condition is likely terminal.	How would you go about discussing potential life extending or pain relieving treatment options given that there is a lower chance of full recovery for this patient? For each, slide the marker to indicate how strongly you feel (0=should not happen; 100=should definitely happen) that you should take the action during the encounter.	Sadness, Urgency

Table S2. Response options and their mappings. (Alphabetical, but presented to participants in random order)

Response	Communication	Empathy	Strategy	Tactics	Calmness	Comfort	Matter-of-factness	Explanation	Education	Control	Caregiver Engagement?	Engagement	Reliance on Others?	Simplification	Thoroughness
…are assertive with {the patient / you} to make sure {they provide / you are able to elicit} all necessary details			✓	✓		✓		✓	✓						✓
…are calm, patient, reassuring, and/or gentle throughout the interaction			✓			✓		✓			✓				
…are insistent with {the patient / you} that {your/their}							✓	✓	✓		✓	✓			✓

Table S2. Response options and their mappings. (Alphabetical, but presented to participants in random order)

Response	Communication	Empathy	Strategy	Tactics	Calmness	Comfort	Matter-of-factness	Explanation	Education	Control	Caregiver Engagement?	Engagement	Reliance on Others?	Simplification	Thoroughness
recommendation is in {the patient's / your} best interests															
…are straightforward and matter-of-fact throughout the interaction	✓						✓	✓	✓		✓			✓	✓
…ask {the/your} caregiver to calm or comfort {the patient / you}					✓	✓	✓	✓							
…calm or comfort {the patient / you}		✓				✓							✓		✓
…demonstrate to {the patient / you}	✓	✓	✓	✓											
…discuss only the affordable treatment options with {the patient / you}	✓	✓	✓	✓			✓	✓				✓		✓	✓
…empathize with {the patient / you} to make {the patient / you} more comfortable		✓	✓	✓	✓	✓				✓	✓	✓	✓		✓
…explain to {the patient / you} in a simplified manner, providing only the			✓	✓			✓		✓		✓			✓	

Table S2. Response options and their mappings. (Alphabetical, but presented to participants in random order)

Response	Communication	Empathy	Strategy	Tactics	Calmness	Comfort	Matter-of-factness	Explanation	Education	Control	Caregiver Engagement?	Engagement	Reliance on Others?	Simplification	Thoroughness
necessary basics															
…explain to {the patient / you} in a thorough manner, providing a great deal of information	✓			✓	✓							✓	✓		✓
…give {the patient / you} educational reading material to take home			✓	✓				✓	✓						
…give {the patient / you} information about who to contact in case of a complication				✓			✓		✓			✓	✓		
…give {the patient / you} some time alone with {the/your} caregiver and return after a few minutes		✓			✓						✓		✓		
…involve {the/your} caregiver in the conversation	✓	✓				✓				✓	✓		✓	✓	✓
…proceed through the interaction deliberately and thoroughly to ensure a deeper understanding		✓			✓		✓			✓			✓	✓	✓
…proceed through the	✓	✓	✓						✓	✓		✓		✓	

Table S2. Response options and their mappings. (Alphabetical, but presented to participants in random order)

Response	Communication	Empathy	Strategy	Tactics	Calmness	Comfort	Matter-of-factness	Explanation	Education	Control	Caregiver Engagement?	Engagement	Reliance on Others?	Simplification	Thoroughness
interaction ignoring the difficult elements of the situation															
…rely on another healthcare provider to handle {the/your} patient education				✓				✓	✓				✓	✓	
…talk only to {the/your} caregiver throughout the interaction				✓		✓		✓			✓		✓	✓	
…talk to {the patient / you} and provide all treatment options			✓		✓		✓	✓				✓	✓		
…talk to {the patient / you} and provide an overview of the process							✓		✓		✓	✓			✓
…talk to {the patient / you} and provide only the best treatment options so as not to overwhelm {the patient / you}	✓	✓			✓				✓			✓		✓	
…talk to {the patient / you} to make {the patient / you} more comfortable	✓				✓	✓						✓			✓
